# Preventing Crystal Agglomeration of Pharmaceutical Crystals Using Temperature Cycling and a Novel Membrane Crystallization Procedure for Seed Crystal Generation

**DOI:** 10.3390/pharmaceutics10010017

**Published:** 2018-01-24

**Authors:** Elena Simone, Rahimah Othman, Goran T. Vladisavljević, Zoltan K. Nagy

**Affiliations:** 1 School of Food Science and Nutrition, University of Leeds, Leeds LS2 9JT, UK; e.simone@leeds.ac.uk; 2 Department of Chemical Engineering, Loughborough University, Loughborough LE11 3TU, Leicestershire, UK; eimah_othman@yahoo.com (R.O.); G.Vladisavljevic@lboro.ac.uk (G.T.V.); 3 School of Bioprocess Engineering, Universiti Malaysia Perlis, Kompleks Pusat Pengajian Jejawi 3, Arau 02600, Perlis, Malaysia; 4 School of Chemical Engineering, Purdue University, West Lafayette, IN 47907-2100, USA

**Keywords:** agglomeration, membrane crystallization, temperature cycling, seeded crystallization

## Abstract

In this work, a novel membrane crystallization system was used to crystallize micro-sized seeds of piroxicam monohydrate by reverse antisolvent addition. Membrane crystallization seeds were compared with seeds produced by conventional antisolvent addition and polymorphic transformation of a fine powdered sample of piroxicam form I in water. The membrane crystallization process allowed for a consistent production of pure monohydrate crystals with narrow size distribution and without significant agglomeration. The seeds were grown in 350 g of 20:80 *w*/*w* acetone-water mixture. Different seeding loads were tested and temperature cycling was applied in order to avoid agglomeration of the growing crystals during the process. Focused beam reflectance measurement (FBRM); and particle vision and measurement (PVM) were used to monitor crystal growth; nucleation and agglomeration during the seeded experiments. Furthermore; Raman spectroscopy was used to monitor solute concentration and estimate the overall yield of the process. Membrane crystallization was proved to be the most convenient and consistent method to produce seeds of highly agglomerating compounds; which can be grown via cooling crystallization and temperature cycling.

## 1. Introduction

Crystal agglomeration is a common phenomenon for many chemical and pharmaceutical compounds. It is usually undesirable since the formation of agglomerates promotes the entrapment of mother liquid and, therefore, compromises the purity of the dried product. Furthermore, particle agglomeration can generate broad crystal size distributions (CSD) and induce the formation of a large amount of fines during storage and transport [1,2].

The effect of operating parameters on crystal agglomeration during crystallization processes has been studied via both experimental and modelling work [2,3]. The main variables affecting agglomeration were found to be solid content in suspension, particle size, stirring rate, and supersaturation at seeding or nucleation. Furthermore, both solvent and solute physical and chemical characteristics were found to strongly affect this phenomenon [1,4,5]. Recently, the effect of particle morphology on crystal agglomeration was studied using a novel image analysis routine [6]. An opportune choice of solvent, careful seeding and temperature cycling can largely decrease the degree of agglomeration. In fact, repeated cycles of heating and cooling were found to promote de-agglomeration [7].

In some cases, the formation of regular agglomerates (mostly spherical) is desired, since they are easy to handle during downstream operations and can be more efficiently compacted [8]. Spherical agglomerates can be formed using a bridging liquid [9,10], a specific additive [11] or by cooling an oil-in-water emulsion where the crystallizing solution is localized within oil droplets [12]. Spherical agglomerates have recently been produced successfully in a continuous MSMPR (mixed suspension, mixed product removal) reactor [12] and in a microfluidic device [13].

Large agglomerates of poorly water soluble drugs can also be obtained in the form of crystanules, particles with mixed characteristics of both crystals and granules. These are produced by melting the insoluble drug and forming an emulsion consisted of drug droplets in water. The droplets are then solidified, by cooling, in the form of large agglomerates. The addition of a polymeric additive can improve the mechanical properties of these crystanules [14,15,16].

Membrane crystallization is a relatively new technique based on the use of a porous material as a semi-permeable barrier between two phases. The membrane can be used to create supersaturation by solvent evaporation, antisolvent or reactant addition, and mixing with a colder solvent [17,18]. The first membrane crystallization process dates back to 1917 when Kober used a polymeric membrane to evaporate water from aqueous solutions of ammonium sulfate and hydrochloric acid, and precipitated salts upon increasing the solution supersaturation [18]. Renewed interest in membrane crystallization aroused in the 80’s when microporous membranes for water treatment became popular [19]. Membrane distillation and reverse osmosis processes were proposed and applied [20,21]. More recently, membranes were used to crystallize proteins and macromolecules [22,23,24]. The presence of a membrane adds a supplementary resistance to mass transfer, but it also offers additional control over the nucleation kinetics (the nature of the membrane can significantly change the surface tension of the crystallized material) and polymorphic outcome of crystallization [25,26,27].

In this work, a flat isoporous nickel membrane installed in a stirred cell was used to produce micro-seeds of piroxicam monohydrate by reverse antisolvent addition. Piroxicam is a non-steroidal anti-inflammatory drug; it can crystallize in two different polymorphs or as monohydrate in the presence of water. This last form is characterized by high tendency of agglomeration. The membrane crystallization procedure used in this work allows producing non-agglomerated crystals of piroxicam monohydrate with narrow CSD, which can easily be filtered and dried [28,29], or used as seeds while still in slurry. The use of the membrane guarantees a higher polymorphic purity, lower tendency to agglomeration, and a narrower crystal size distribution compared to traditional batch crystallization techniques (cooling, antisolvent or reverse antisolvent addition) [28,29]. Seed crystals produced by membrane crystallization were compared to those prepared using traditional batch techniques. Furthermore, a specific cooling profile with temperature cycling was determined and applied in order to reduce agglomeration and promote crystal growth of the seed crystals. The experimental results show that a combination of specifically tailored membrane crystallization seeds and temperature cycling can efficiently reduce agglomeration and promote crystal growth of piroxicam monohydrate.

## 2. Materials and Methods

**Figure 1:**
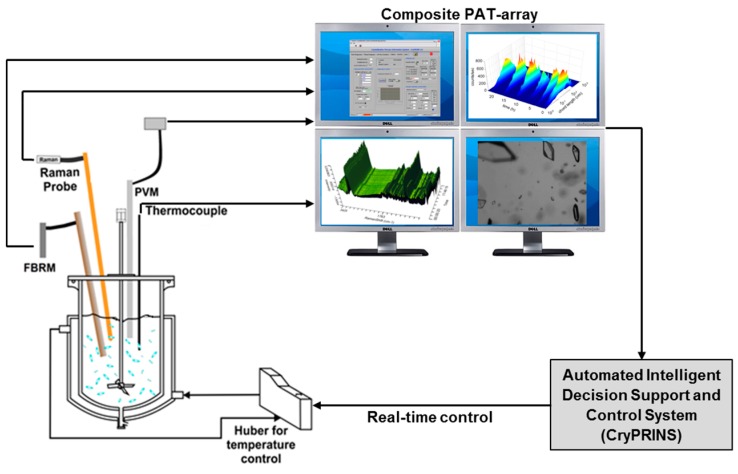
Schematic of the rig used for the experiments.

Piroxicam (99% purity) was purchased from Hangzhou Hyper Chemicals Limited (Hangzhou, China). Acetone (99.98% purity) purchased from Fisher Scientific (Loughborough, UK) and de-ionized water (Millipore ultra-pure system) were used as solvent and antisolvent for the drug. A 400 mL jacketed glass vessel was used to carry out the 350 g cooling crystallization experiments. The vessel was equipped with an overhead polytetrafluoroethylene (PTFE) pitch blade stirrer (325 rpm was the stirring speed used in all experiments). A schematic of the rig used for the experiments is shown in Figure 1.

A PT-100 temperature probe connected to a Huber Ministat 125 thermoregulator (Huber Kältemaschinenbau AG, Offenburg, Germany) was used to control the internal temperature of the vessel as well as the temperature in the jacket. Several process analytical technology (PAT) tools were used to monitor the experiments, including: (1) a Kaiser RXN1 Raman analyzer with immersion probe and 785 nm laser, equipped with iC Raman 4.1 software, (2) a Mettler-Toledo particle vision and measurement (PVM) V819 probe with an on-line image acquisition software (version 8.3), and (3) a G400 Mettler-Toledo Focused Beam Reflectance Measurement (FBRM) probe equipped with iC FBRM version 4.3 software. Data was processed using MatLab R2015, iC Raman 4.1 and Excel 2013. Additionally, the CryPRINS software (Crystallization Process Informatics System, version 2.0), which allows real time temperature control and simultaneous monitoring of signals from different probes (FBRM, ATR-UV/Vis, thermocouple, conductivity probes and pH-meter), was used.

### 2.1. Seeded Batch Crystallization Experiments

The choice of solvent for the seeded cooling crystallization experiments was made based on available solubility data and past literature on piroxicam crystallization [30,31]. In order to nucleate and grow crystals of piroxicam monohydrate, a mixture of water and an organic solvent needs to be used. The addition of an organic solvent is necessary to avoid an excessive reduction of piroxicam solubility in the final solvent mixture, since this compound is almost insoluble in pure water. Acetone was selected as the organic solvent for this work, and a 20:80 *w/w* water-acetone mixture was used as solvent in the experiments presented here [30]. In such solvent mixture, the piroxicam solubility still increase considerably as temperature rises, which is a necessary condition to increase the yield of cooling crystallization processes.

Solutions of piroxicam with concentration of 126 mg/g solvent (saturation temperature of 40 °C) were prepared and heated up to 50 °C for 30 min in order to fully dissolve the solid piroxicam. After that, solutions were cooled down to 37 °C and seed crystals were added. In order to reduce crystal agglomeration immediately after seeding, the amount of seed crystals added to the solution was set to 2% of the total mass of piroxicam dissolved. An additional experiment using 6% seed crystals was performed for comparison, using crystals made by membrane crystallization. Piroxicam monohydrate was found to have very slow kinetics of nucleation and growth; for this reason a specific temperature profile was designed for the seeded experiments in order to maximize the recovery yield of piroxicam by allowing, at the same time, growth and secondary nucleation. After seeding, the solution was slowly cooled down to 10 °C (−0.1 °C/min) and then left at that temperature for 10 h. During such period of time the solution supersaturation was completely depleted by growth, secondary nucleation and, partly, by agglomeration. After the isothermal period, in order to remove the fine crystals generated by secondary nucleation and reduce agglomeration, temperature cycling was applied.

Slurries underwent nine temperature cycles of 20 °C amplitude with heating/cooling rate of ±0.2 °C/min. The cycles’ amplitude and rates were chosen, based on preliminary experiments (see Supplementary Materials Figures S1 and S2), in order to accelerate the dissolution of fines and de-agglomeration [29].

In addition to the use of fixed amplitude cycles, direct nucleation control (DNC) was also tested. The details of this control strategy are described elsewhere [7,32,33,34,35]. Experimental results for the two DNC experiments performed are shown in the Supplementary Materials Figures S8–S11).

### 2.2. Seeds Preparation

Seed crystals were prepared using membrane crystallization, antisolvent crystallization, and polymorphic transformation, as described in the following sections. Cooling crystallization with in situ nucleation was also performed but seed crystals could not be produced in a reasonable batch time because of the very slow kinetics of primary nucleation for piroxicam monohydrate.

#### 2.2.1. Membrane Crystallization

**Figure 2:**
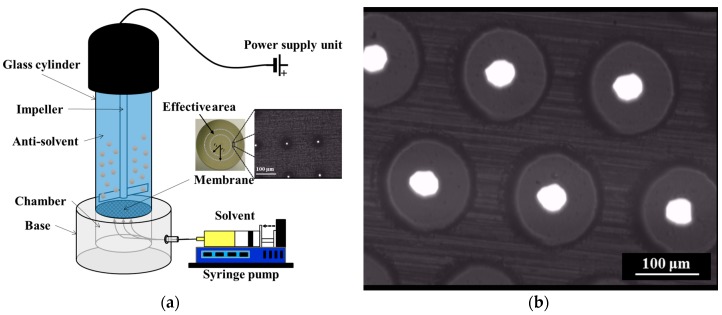
(**a**) Schematic of the rig used for membrane crystallization; (**b**) Scanning electron microscope (SEM) image of the Ni membrane used for the experiments.

Piroxicam microcrystals were prepared by reverse antisolvent addition using a flat, disc-shaped membrane installed in a stirred cell, as shown in Figure 2a. The cell and membranes were purchased from Micropore Technologies Ltd. (Derby, UK). This apparatus is normally used for membrane emulsification, a gentle process for the formation of droplets and particles in the micron range [36,37,38].

The rotation speed of the stirrer was fixed to 1500 rpm, corresponding to a peak shear stress at the membrane surface of 17.5 Pa. A 24 V direct current (DC) motor (Instek model PR-3060, New Taipei City, Taiwan) was used to drive the stirrer. The nickel membrane used for the experiments had an operative area of 8.55 cm^2^ and an effective diameter of 3.3 cm. It presented ≈24,690 pores with diameter of 40 μm, arranged hexagonally and spaced apart at a constant distance of 200 μm, as shown in the scanning electron microscope (SEM, Hitachi Ltd., Tokyo, Japan) image of Figure 2b.

The membrane was placed at the bottom of the cell which was filled with 30 mL of deionized water. A solution containing 15 g·L^−1^ of piroxicam in acetone was injected through the membrane using a syringe pump (Harvard Apparatus model 11 Elite, Harvard Apparatus, Holliston, United States). The feed flow rate through the membrane was 18 mL·min^−1^, corresponding to a transmembrane flux of 4000 L m^−2^·h^−1^). The total volume of the feed solution injected was 6 mL and the volume mean diameter of microcrystals was between 25–35 μm, as measured by Malvern Mastersizer 2000 (with a Hydro 2000SM dispersant unit, Malvern Instruments Ltd., Malvern, UK) [29]. In this reverse antisolvent crystallization procedure, the membrane acts as a physical support for the generation and sustenance of a controlled supersaturation for the nucleation and growth of the crystals. The feed is injected into the aqueous phase through multiple equally spaced injection microjets where individual crystals can nucleate and grow before being transferred to the bulk as the result of the shear stress created by the stirrer. This procedure allows the production of crystals with very narrow CSD, as well as low agglomeration tendency. Crystals smaller than 25 μm can be obtained with the same apparatus [28], but the 25–35 μm size range was chosen to minimize particle agglomeration immediately after seeding. After each experiment, the membrane was sonicated in acetone for 30 min, washed with deionized water in an ultrasonic bath for 5 min and then stored in acetone. The slurry containing the monohydrate microcrystals was transferred from the cell to a beaker where crystals were allowed to settle at the bottom. Crystals were then collected with a pipette and transferred directly to the vessel for the seeded batch experiments. A small amount of sample was used to measure the CSD using the Malvern Mastersizer 2000 previously described. A saturated solution of piroxicam monohydrate in water at room temperature was used as dispersant.

#### 2.2.2. Antisolvent Crystallization

Seed crystals from antisolvent crystallization were produced by pumping water with a peristaltic pump (Masterflex L/S digital drive, Masterflex Technical Hoses Limited, Oldham, UK) at a rate of 2.5 mL/min into a solution of piroxicam in acetone with concentration of 35 mg/g of solvent. The solution was prepared by dissolving solid piroxicam at 40 °C in the 400 mL jacketed vessel. The rate of antisolvent was chosen after a few preliminary experiments in order to obtain pure monohydrate piroxicam at the end of the batch. In fact, nucleation of form II of piroxicam was observed using both faster and slower cooling rates. During the experiments, FBRM and PVM were used to monitor nucleation, agglomeration and shape of the particles. The Malvern Mastersizer 2000 previously described was used to measure the CSD of the crystals obtained at the end of the experiments (after filtration and drying).

#### 2.2.3. Polymorphic Transformation

Piroxicam form I (fine powder as purchased) was suspended in water and left to transform to monohydrate. A total amount of 2.2 g of piroxicam was suspended in 500 g of water at 20 °C. Raman spectroscopy (ThermoFisher Scientific, Waltham, MA, USA) was used to monitor, in situ, the polymorphic transformation of form I into piroxicam monohydrate. After transformation, crystals were filtered and dried, and their CSD was measured by the Malvern Mastersizer previously described. 

#### 2.2.4. Cooling Crystallization

Solutions of piroxicam in a 20:80 *w*/*w* water-acetone mixture were prepared in the 400 mL vessel, as described in the Equipment section.

Solutions containing 6.8, 7, 8.8, 11.4, and 14 mg of piroxicam per 1 g of solvent were heated up until the drug was completely dissolved and then cooled down to 5 °C at a cooling rate of 0.5 °C/min. Nucleation of monohydrate crystals was not detected either during the cooling profile or after leaving the solution at 5 °C for few hours, indicating very slow primary nucleation kinetics. In conclusion, piroxicam monohydrate seeds could not be produced in a reasonable batch time by cooling crystallization with the equipment available, because of the extremely slow primary nucleation kinetics.

## 3. Results and Discussion

### 3.1. Seeds Characterization and Comparison

**Figure 3:**
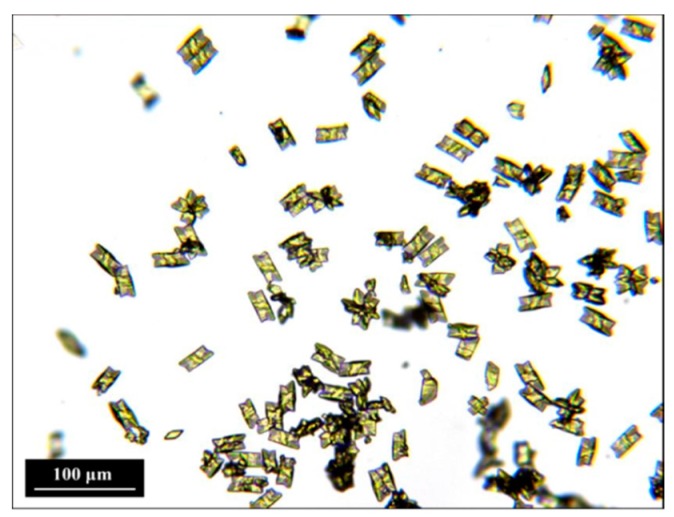
Monohydrate crystals produced by membrane crystallization.

Seeds obtained by membrane crystallization are shown in Figure 3. Crystals displayed the characteristic yellow color of piroxicam monohydrate (polymorphic purity was checked with Raman microscopy and differential scanning calorimetry) and the shape of a prism with a rhombus base.

**Figure 4:**
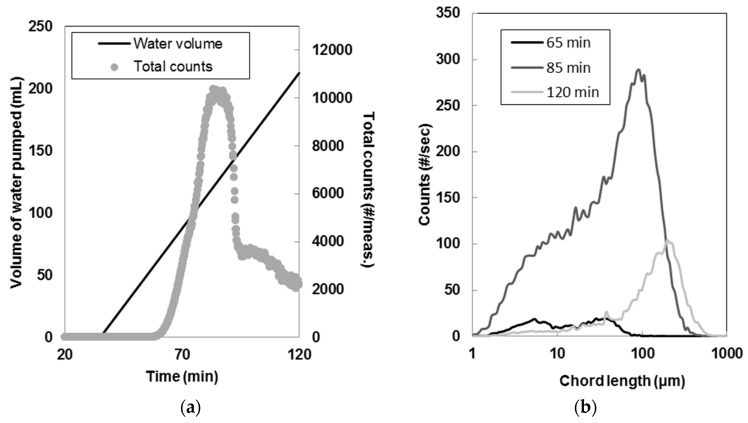

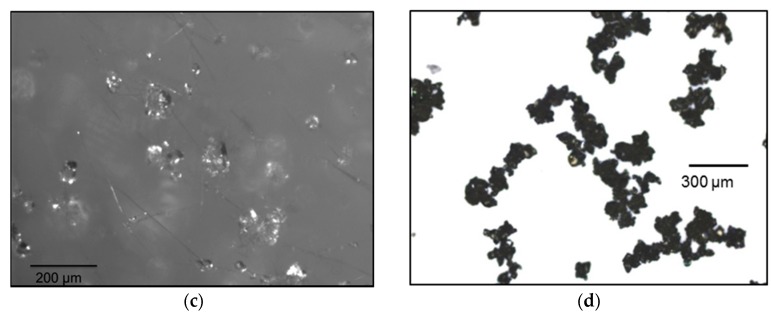
(**a**) Total counts and volume of water pumped in the vessel during antisolvent crystallization of piroxicam (2.5 mL/min water flow); (**b**) Evolution of the chord length distribution during the experiment; (**c**) particle vision and measurement (PVM) image of the crystals during nucleation and growth. Needle-shape crystals were identified as piroxicam form II; (**d**) Crystals after filtration and drying. Form II was not detected at the end of the experiment.

These seeds did not show significant agglomeration and they were characterized by a volume weighted mean diameter measured using the Mastersizer in a range of 25–35 μm. Figure 4d shows the seeds obtained by antisolvent crystallization; crystals were highly agglomerated and it was difficult to identify their shape. Raman microscopy confirmed that crystals were pure piroxicam monohydrate after filtration and drying. However, Figure 4c shows a PVM image taken after nucleation where several monohydrate agglomerates were observed together with needle-shaped crystals of piroxicam form II. Most likely, a mixture of monohydrate and form II was nucleated and then converted to the stable monohydrate during the batch.

The sudden drop in total counts/measurement recorded by the FBRM (Figure 4a,b) was probably due to the change in crystal shape associated to the polymorphic transformation (from needle-shaped form II to cubic piroxicam monohydrate). The formation of monohydrate agglomerates might also have contributed to the decrease in the total counts/measurement.

**Figure 5:**
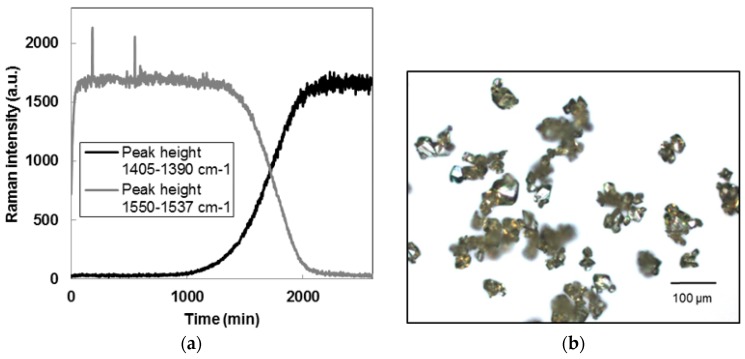
(**a**) Raman signal during polymorphic transformation from form I piroxicam to monohydrate; (**b**) Microscopic image of the monohydrate crystals obtained at the end of the experiment.

Figure 5b shows the monohydrate crystals obtained at the end of the polymorphic transformation from form II to piroxicam monohydrate, in water. The transformation took almost two days and generated fine monohydrate crystals that were considerably agglomerated.

**Figure 6:**
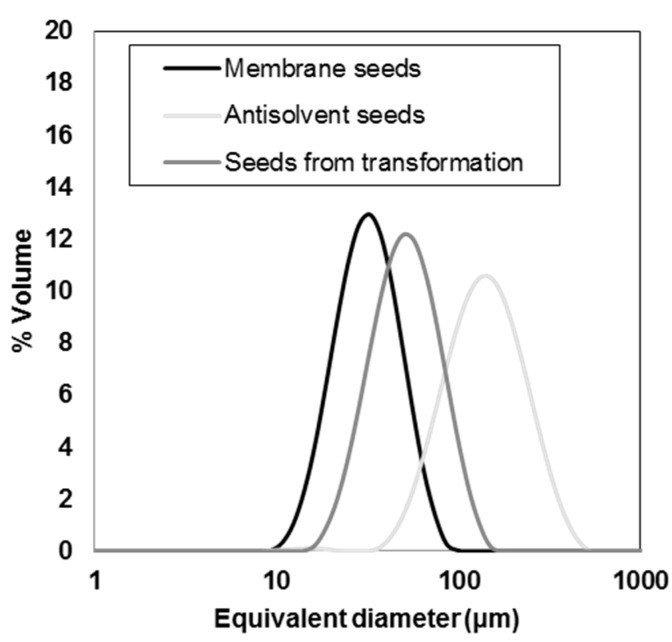
Crystal size distribution of the different seeds from Malvern Mastersizer.

Figure 5a shows the intensity of two different Raman peaks, after second derivative and smoothing calculation. The peak at 1405–1390 cm^−1^ was associated with solid piroxicam monohydrate, while the peak at 1550–1537 cm^−1^ was typical of form I. During the polymorphic transformation, the intensity of the form I peak decreased, while the peak intensity for the monohydrate increased. The polymorphic transition was also detectable qualitatively by a change of color of the slurry from white (form I) to bright yellow (monohydrate). The Malvern Mastersizer was used to determine the crystal size distribution of the three types of seeds. Seeds produced by membrane crystallization were analyzed in form of slurry, while seeds from antisolvent addition and polymorphic transformation were filtered and dried before being dispersed in a saturated aqueous solution of piroxicam monohydrate. Figure 6 shows the CSD of the three types of seeds used for this work. Membrane crystallization generated narrower CSDs than antisolvent addition.

Some significant statistics calculated with the Malvern software are shown in Table 1. Membrane seeds have the lower span (calculated as [d(0.9) − d(0.1)]/d(0.5)] and, therefore, related to the width of the CSD) and the lower volume weighted mean diameter. Furthermore, as shown in the optical micrographs membrane seeds are less agglomerated compared to the polymorphic transformation and antisolvent ones.

### 3.2. Seeded Growth Experiments

**Figure 7:**
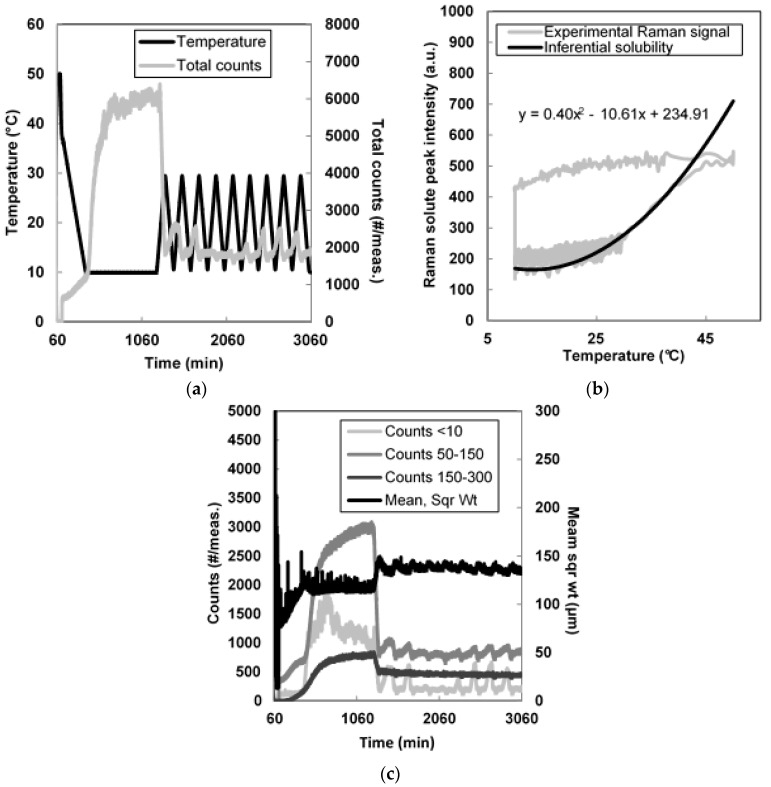
(**a**) Total counts and temperature during the seeded experiments with 2% membrane crystals; (**b**) Intensity of the Raman peak corresponding to dissolved piroxicam (solute); (**c**) Trends for FBRM (Focused Beam Reflectance Measurement) counts and mean of the square weighted chord length distribution.

Similar seeded batch cooling crystallization experiments were carried out using the three types of seed crystals prepared. Solutions saturated at 40 °C were prepared by dissolving solid piroxicam in the chosen solvent at 50 °C. Seeds were added after cooling down to 37 °C, and then the solutions were further cooled down to 10 °C at a rate of −0.1 °C/min. In order to fully deplete supersaturation, the temperature was kept constant at 10 °C. Raman spectroscopy was used to monitor the evolution of solute concentration. The peak intensity at 1443–1438 cm^−1^ (after calculation of second derivative and smoothing) was used as an indication of the amount of piroxicam dissolved in solution. An inferential solubility curve was determined by measuring the values of intensity of such Raman peak for several saturated solutions of piroxicam monohydrate, at different temperatures. A polynomial function was used to interpolate the data and is shown in Figure 7b. The solution supersaturation was consumed by growth, secondary nucleation and, partly, by agglomeration in about 8–10 h, indicating slow kinetics of growth and nucleation. After that, temperature cycling was applied to reduce crystal agglomeration and dissolve fine crystals. A total of nine cycles of 20 °C amplitude was used.

The evolution of the total counts/measurement during the experiment is shown in Figure 7a while Figure 7c shows the most significant FBRM statistics. It can be noticed that after two cycles a stable oscillatory trend for the total counts/meas. and mean of the squared weighted chord length distribution was reached. As shown in Figure 7b, the solute concentration during cycling remained close to equilibrium conditions, indicating a high yield at the end of the experiment.

The trends of the counts/meas. between 50–150 and 150–300 μm (corresponding to the size of monohydrate agglomerates) also reached a stable trend after two cycles. Microscopic images of the crystals from filtered and dried samples (Supplementary Materials Figure S3) also showed a decrease in the number of agglomerates while cycling.

**Figure 8:**
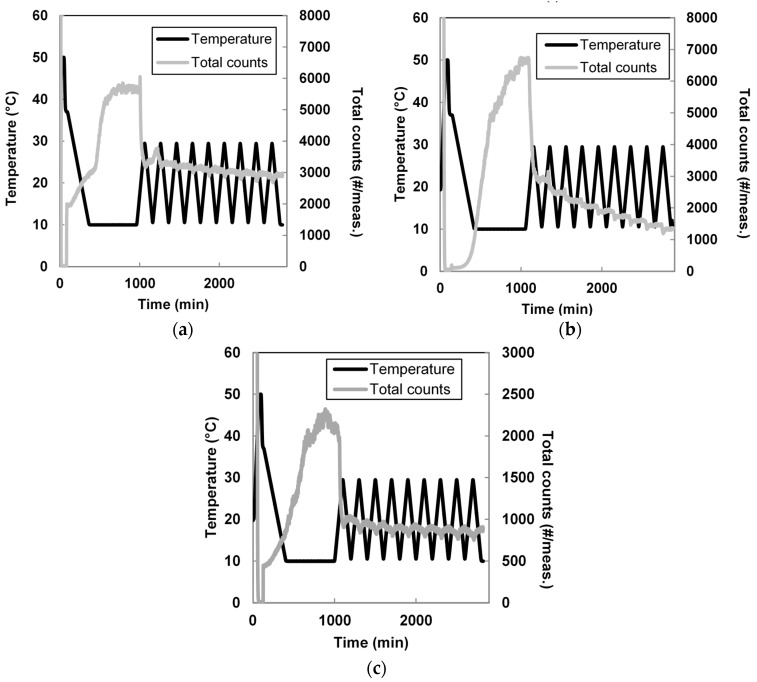
(**a**) Total counts and temperature profile for the seeded experiment with 6% membrane seeds; (**b**) 2% antisolvent addition seeds; (**c**) polymorphic transformation seeds.

Figure 8 shows the trends for the total counts/measurement for the other three seeded cooling and cycling experiments. In all cases, a longer time was needed to reach a stable oscillating trend in the total counts/measurement compared to the seeded experiments with 2% membrane seeds. In fact, nine cycles were not enough to reach a stable oscillating trend for the antisolvent addition and 6% membrane seeds, as shown in Figure 8a,b. Figure 8c shows that in the case of the polymorphic transformation seeds, four temperature cycles were needed to reach a stable trend. The maximum amount of total counts reached after 10 h at 10 °C for this last type of seeds was around 2100 total counts/measurement, significantly lower than the maximum reached for all the other experiments (between 5000 and 7000 total counts/measurement).

**Figure 9:**
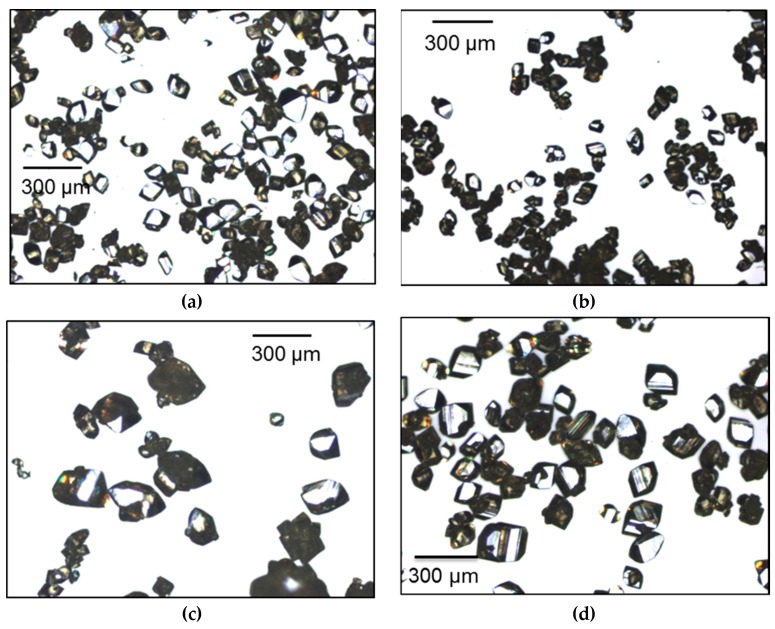
Microscopic images of crystals grown from: (**a**) membrane seeds (2% of the total mass of piroxicam); (**b**) membrane seeds (6% of the total mass of piroxicam); (**c**) antisolvent seeds; (**d**) transformation seeds.

In order to better compare the crystals obtained at the end of each cycling experiments, microscopic images are shown in Figure 9. The narrowest CSDs were obtained with membrane seeds and seeds from polymorphic transformation, as it can be noted in Figure 9a,b,d. The final degree of agglomeration at the end of each experiment was very similar, as shown in Figure 9. However, in Figure 9c few large agglomerates could still be identified in the crystals obtained using antisolvent seeds. Microscopic images of crystals collected at different times during the seeded crystallization experiments can be found in the Supplementary Materials Figures S3–S7.

**Figure 10:**
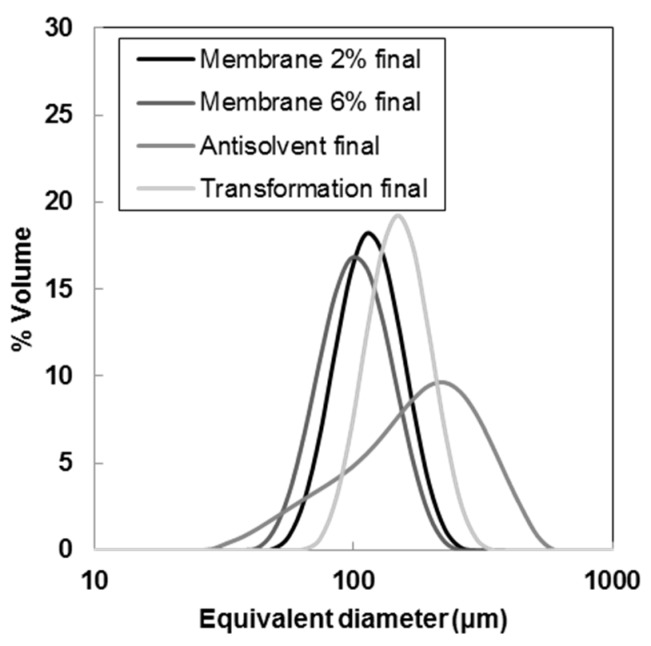
Crystal size distributions of the crystals obtained at the end of the seeded experiments. Crystal size distributions (CSDs) measured with the Malvern Mastersizer.

The CSDs and the main statistics for the crystals obtained at the end of the cycling experiments are shown in Figure 10 and Table 2. The highest increase in volume weighted mean diameter during the cycling experiment was observed for the membrane seeds: +243% when 2% seeds were used, and +205% for 6% seeds.

A decrease in the span of the distribution (parameter related to its narrowness) was noticeable for all seeds apart from the ones obtained by antisolvent addition. In particular, the polymorphic transformation seeds led to the lowest value of the span and its highest decrease. The final crystal size distributions for each of the cycling experiments are shown in Figure 10.

While the membrane seeds and the ones from polymorphic transformation generated narrow and unimodal distributions, the antisolvent seeds led to a very broad CSD with a high amount of fines. In conclusion, using membrane seeds and polymorphic transformation seeds allowed for a narrow CSD with low tendency to agglomeration. The number of temperature cycles needed to reach the equilibrium size and to remove all agglomerates was around 2 for the membrane seeds (2% of the total mass of piroxicam in solution at the moment of seeding), and 4 for the polymorphic transformation seeds. Therefore, a shorter batch time was needed when membrane seeds were used in the correct amount. Furthermore, in order to produce 2 g of polymorphic transformation seeds, a fine powdered sample of piroxicam form I needed to be suspended in water for over 40 hours, for complete conversion to the monohydrate form. On the other hand, producing the same amount of membrane seeds does not require any previous sample preparation and takes only a few seconds. Additionally, membrane seeds can be produced in a continuous mode with a similar apparatus [28] making this seed production method particularly efficient for piroxicam and other highly agglomerating compounds.

## 4. Conclusions

A novel procedure for seeds production was used in combination with temperature cycling to grow crystals of a highly agglomeration prone compound (piroxicam monohydrate). The membrane seeds were compared to seeds obtained by conventional antisolvent addition and polymorphic transformation. Crystals of monohydrate could not be obtained by cooling crystallization with in situ primary nucleation.

The membrane seeds allowed a narrow crystal size distribution to be obtained at the end of the batch without significant agglomeration. The quality of the final crystals in terms of final crystal size distribution was comparable to the one gained using seeds from a polymorphic transformation. However, membrane seeds can be produced in a faster and more efficient way compared to the polymorphic transformation ones.

Furthermore, membrane seeds were used in combination with the direct nucleation control strategy, which led to large crystals with a narrow crystal size distribution and no visible agglomeration.

In conclusion, this study shows that crystal agglomeration can be prevented efficiently by using a specific seeds preparation and temperature cycling. Agglomeration is an undesired phenomenon since it causes the broadening of the crystal size distribution and solvent incorporation. Membrane crystallization is a fast and efficient way to obtain non-agglomerated crystals with narrow CSD, which can be used as a final product (after filtration and drying) or as seeds when larger crystals are required. As shown by the experimental results, this method is particularly useful for compounds with a slow nucleation rate that cannot be generated at low supersaturation to avoid agglomeration.

## Figures and Tables

**Table 1:** Malvern Mastersizer data for the seeds used, where d(0.1), d(0.5), and d(0.9) are the particle diameters at 10%, 50% and 90% of the cumulative distribution, span was calculated as [d(0.9) − d(0.1)]/d(0.5), and D[4,3] and D[3,2] are the volume and surface weighted mean diameters, respectively. The table shows the average values of all the measurement replicates and corresponding standard deviations.

Seeds Type	d(0.1) (µm)	d(0.5) (µm)	d(0.9) (µm)	Span(-)	D[4,3] (µm)	D[3,2] (µm)
Membrane	19.8 ± 1.23	32.5 ± 1.85	53.1 ± 3.62	1.03 ± 0.07	34.8 ± 4.25	30.2 ± 1.73
Transformation	28.8 ± 0.49	50.6 ± 0.83	87.5 ± 1.45	1.16 ± 0.002	55.0 ± 0.90	46.0 ± 0.78
Antisolvent	65.6 ± 0.05	121 ± 3.39	220.9 ± 12.85	1.28 ± 0.07	134 ± 5.15	101 ± 2.67

**Table 2:** Malvern Mastersizer data for the crystals at the end of the seeded experiments, where d(0.1), d(0.5), and d(0.9) are the particle diameters at 10%, 50% and 90% of the cumulative distribution, Span was calculated as [d(0.9) − d(0.1)]/d(0.5), and D[4,3] and D[3,2] are the volume and surface weighted mean diameters, respectively. The table shows the average values of all the measurement replicates and corresponding standard deviations.

Sample	d(0.1) (µm)	d(0.5) (µm)	d(0.9) (µm)	Span(-)	D[4,3] (µm)	D[3,2] (µm)
*Membrane 2%*	78.7 ± 1.21	115 ± 0.46	166 ± 3.61	0.76 ± 0.04 (−25%)	119 ± 0.9 (+243%)	110 ± 0.33 (+263%)
*Membrane 6%*	67.7 ± 0.31	101 ± 0.70	152 ± 1.60	0.83 ± 0.01 (−19%)	106 ± 0.74 (+205%)	96.7 ± 0.63 (+220%)
*Transformation*	104 ±0.45	148 ± 0.62	211 ± 0.86	0.72 ± 0.001 (−38%)	153 ± 0.64 (+179%)	142 ± 0.60 (+209%)
*Antisolvent*	70.3 ± 1.07	181 ± 1.59	340 ± 1.97	1.49 ± 0.01 (+16%)	195 ± 1.38 (+46%)	138 ± 1.35 (+36%)

## Supplementary Materials

The following are available online at http://www.mdpi.com/1999-4923/10/1/17/s1, Figure S1: Temperature profile and total counts evolution during a preliminary cycling experiments performed in order to establish the best cycles’ amplitude and rate, Figure S2: Microscopic images of crystals during the cycling experiment of Figure S1, Figure S3: Microscopic images of crystals during the cycling experiment 1 (2% membrane seeds), Figure S4: Microscopic images of crystals during the cycling experiment 2 (6% membrane seeds), Figure S5: Microscopic images of crystals during the cycling experiment 3 (antisolvent seeds), Figure S6: Microscopic images of crystals during the cycling experiment 4 (polymorphic transformation seeds), Figure S7: FBRM statistics during cycling experiments 1, 3 and 4, Figure S8: Temperature, total counts, Raman intensity and microscopic images of crystals for the DNC experiment 1, Figure S9: Temperature, total counts, Raman intensity and microscopic images of crystals for the DNC experiment 2, Figure S10: Microscopic images of crystals during the DNC experiment 1, Figure S11: Microscopic images of crystals during the DNC experiment 2, Table S1: Malvern Mastersizer data for the seeds and the crystals at the end of the DNC experiment 2.

Supplementary File 1
