# Supplementary material for: Preventing Crystal Agglomeration of Pharmaceutical Crystals Using Temperature Cycling and a Novel Membrane Crystallization Procedure for Seed Crystal Generation
Source: Pharmaceutics. 2018 Jan 24;10(1):17. doi: 10.3390/pharmaceutics10010017 (PMC5874830; doi:10.3390/pharmaceutics10010017)
Supplement: Supplementary File 1 [file pharmaceutics-10-00017-s001.pdf]

Supporting Information: Preventing Crystal Agglomeration of Pharmaceutical Crystals Using Temperature Cycling and a Novel Membrane Crystallization Procedure for Seed Crystals Generation

Elena Simone, Rahimah Othman, Goran T. Vladisavljević and Zoltan K. Nagy

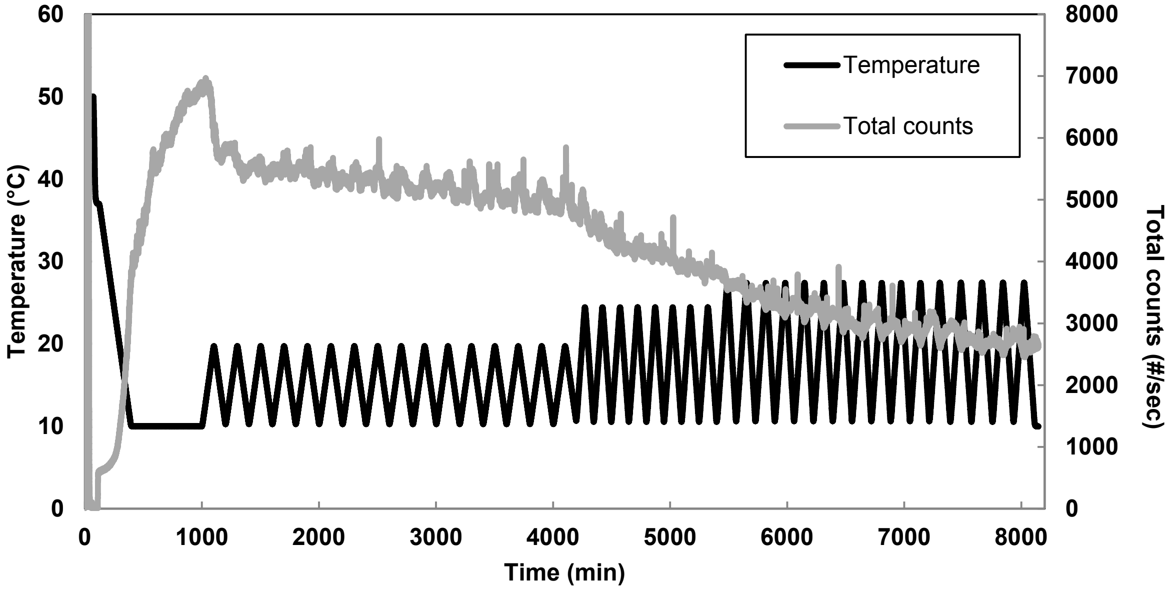

Figure S1. Temperature profile and total counts evolution during a preliminary cycling experiments performed in order to establish the best cycles' amplitude and rate.

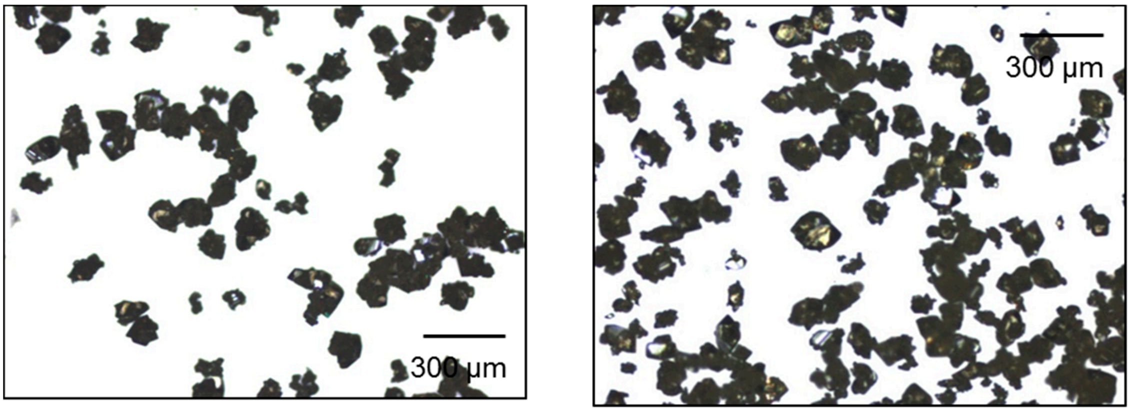

(**a**) (**b**)

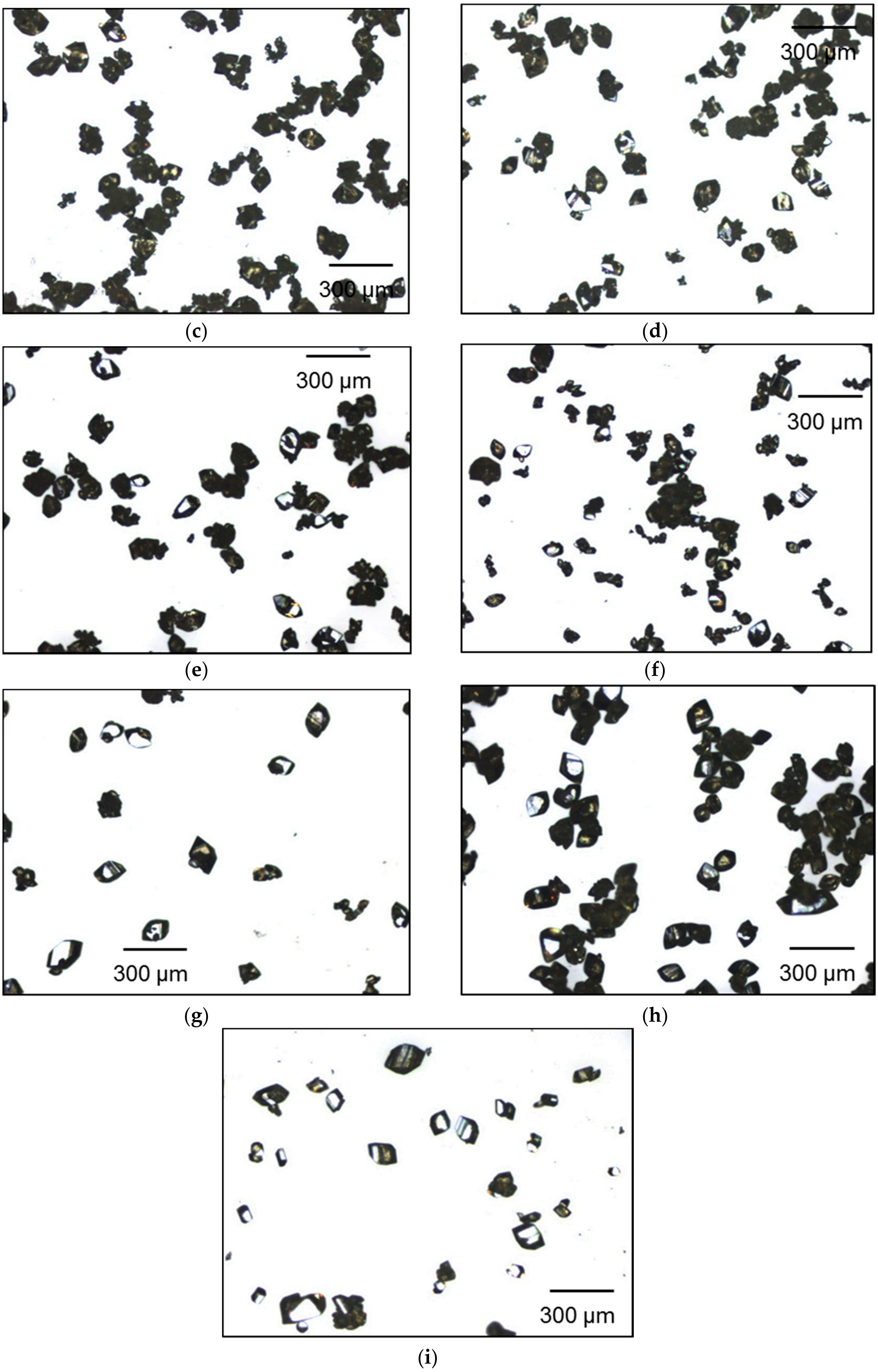

Figure S2. Microscopic images of crystals during the preliminary cycling experiment of Figure S1 taken at times (**a**) 1000 min (**b**) 1380 min (**c**) 2400 min (**d**) 2800 min (**e**) 3950 min (**f**) 4250 min (**g**) 4410 min (**h**) 5410 min (**i**) 7170 min and (**j**) end of experiment.

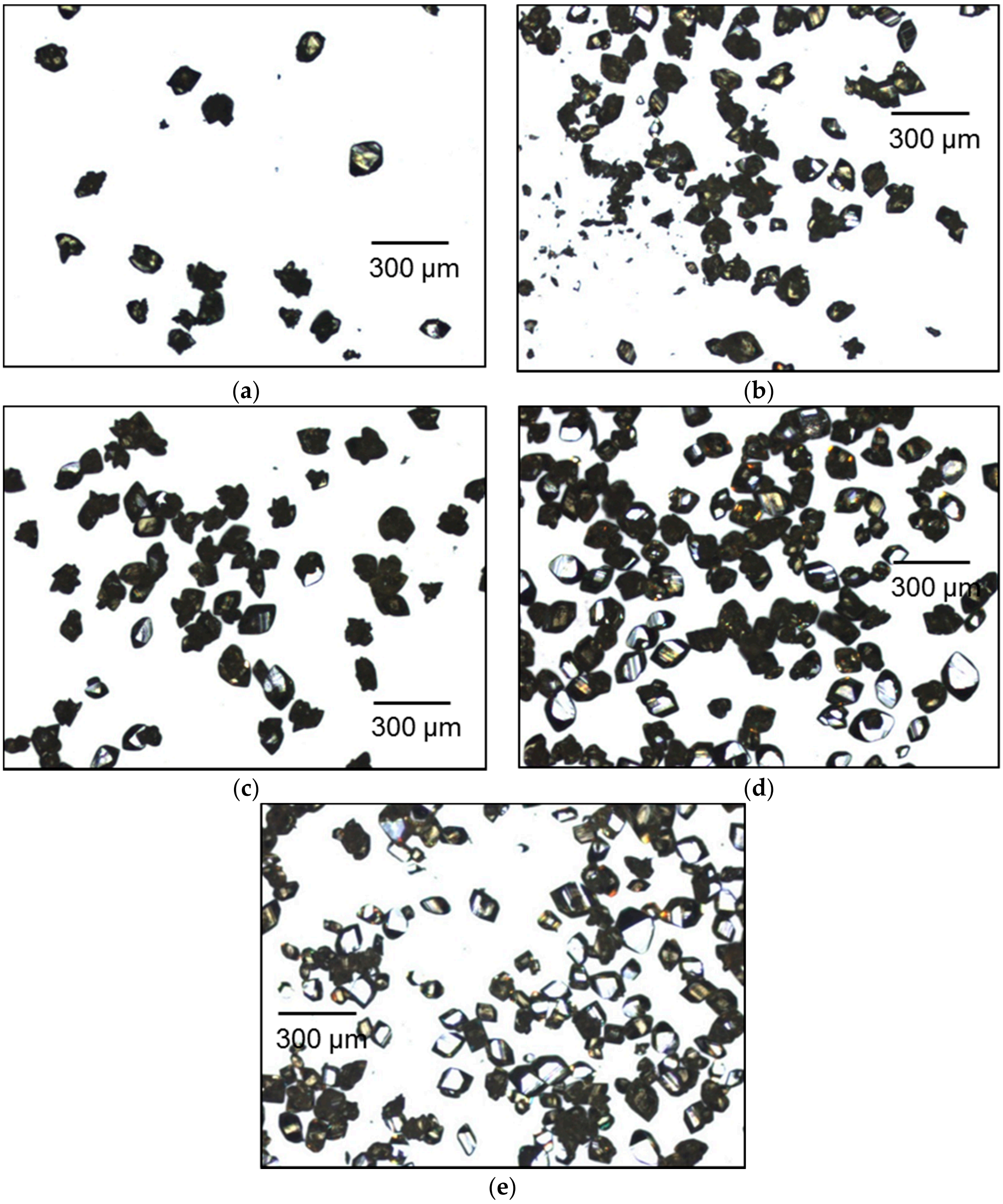

Figure S3. Microscopic images of crystals during the cycling experiment 1 (2% membrane seeds) taken at times (**a**) 1200 min (**b**) 1250 min (**c**) 1590 min (**d**) 2640 min (**e**) 3060 min.

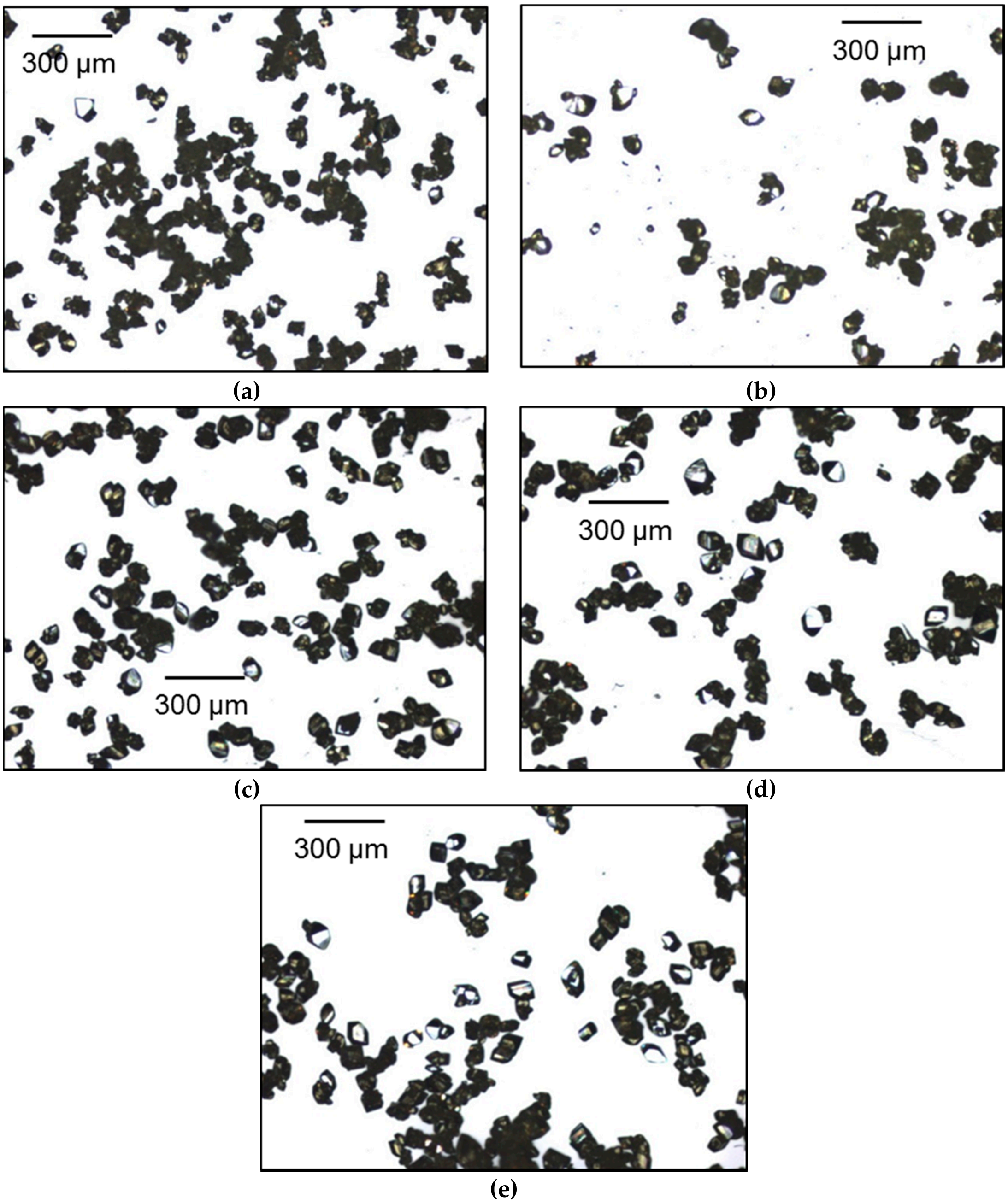

Figure S4. Microscopic images of crystals during the cycling experiment 2 (6% membrane seeds) taken at times (**a**) 340 min (**b**) 1120 min (**c**) 1390 min (**d**) 1610 min (**e**) 2790 min.

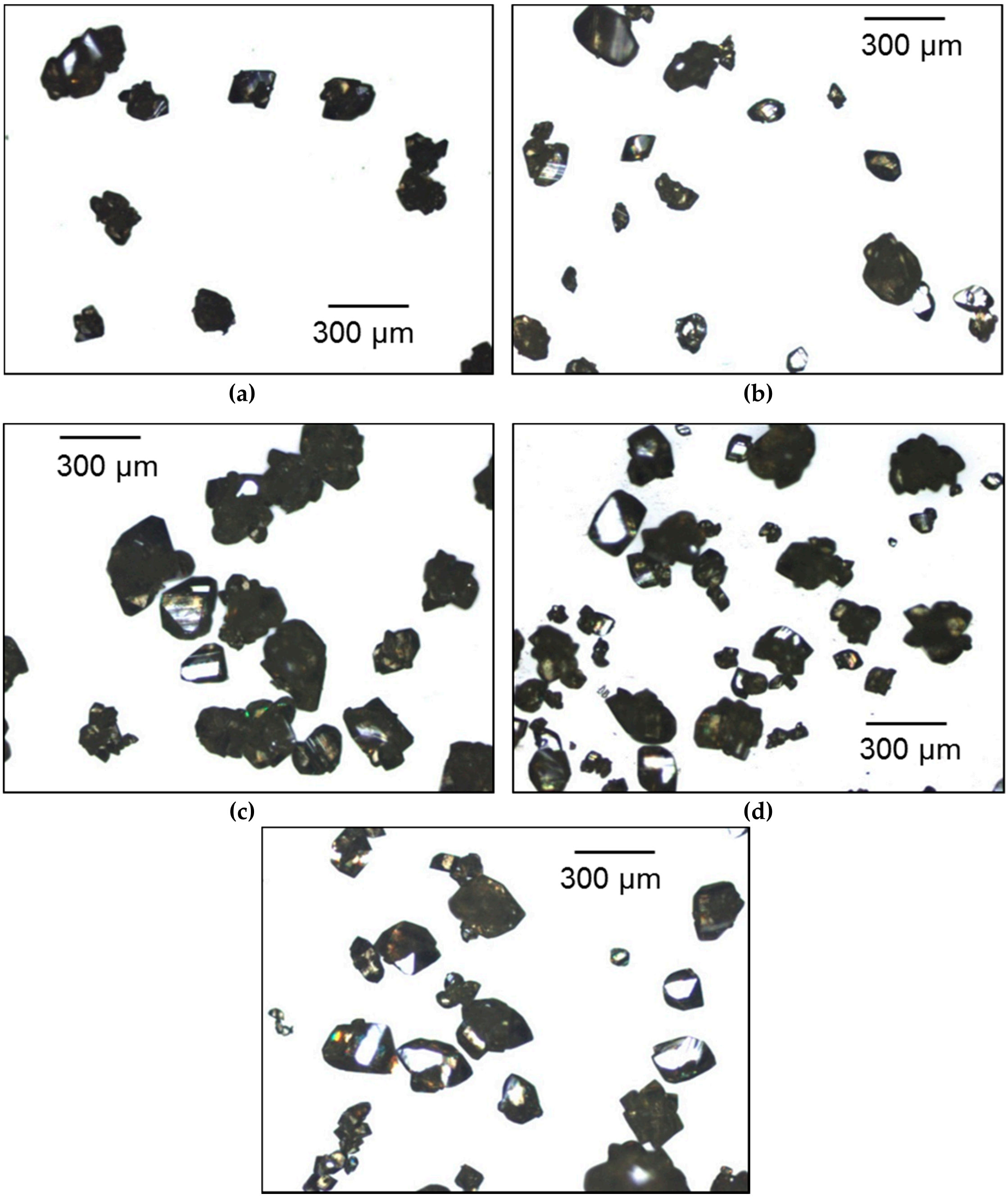

Figure S5. Microscopic images of crystals during the cycling experiment 3 (antisolvent seeds) taken at times (**a**) 445 min (**b**) 1405 min (**c**) 1665 min (**d**) 2020 min (**e**) 2885 min.

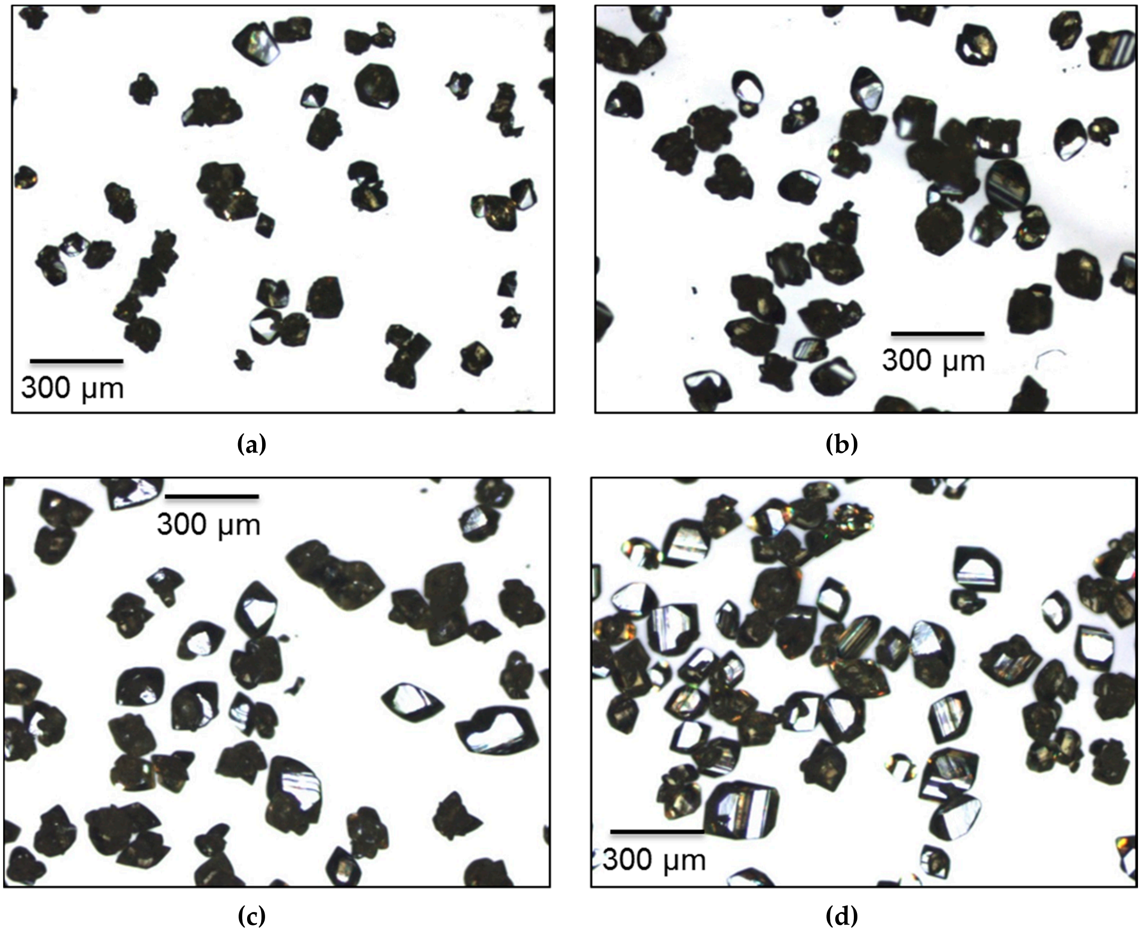

Figure S6. Microscopic images of crystals during the cycling experiment 4 (polymorphic transformation seeds) taken at times (**a**) 407 min (**b**) 1350 min (**c**) 1907 min (**d**) 2825 min.

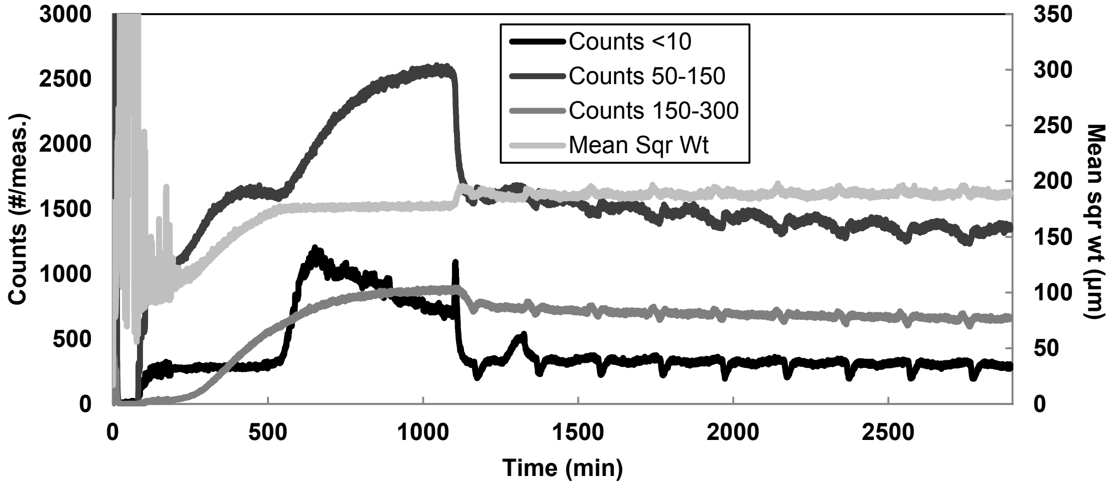

(a)

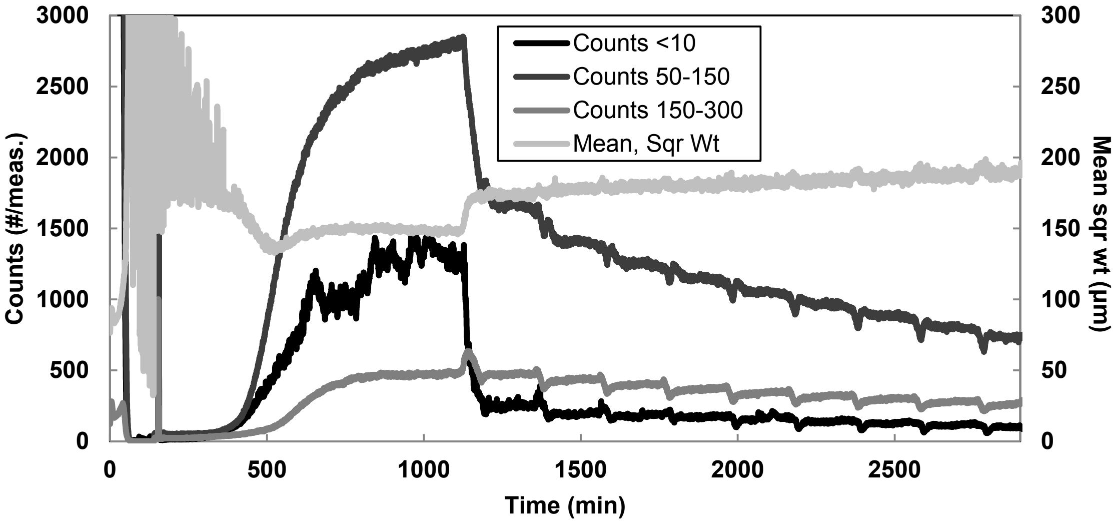

(b)

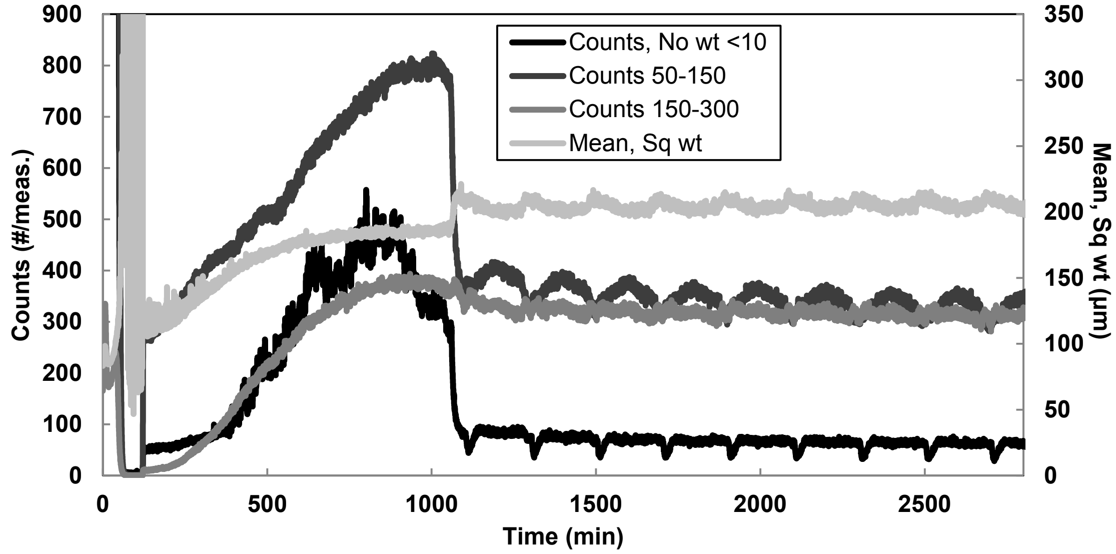

(c)

Figure S7. FBRM statistics during cycling experiments 1, 3 and 4.

Direct nucleation control (DNC) experiments

In the direct nucleation control strategy the FBRM probe is used in combination with CryPRINS to allow the growth of crystals present in the crystallizer and minimize secondary nucleation [7,32–35]. In the two DNC experiments performed in this work ±0.2 °C/min was imposed as heating/cooling rate and 850 and 1000 were selected as total counts/measurement setpoints. Piroxicam monohydrate crystals were seeded with the same procedure described in the previous paragraph. Temperature was decreased from 37 to 10 °C at a rate of 0.1 °C/min and only then the feedback control was started.

The feedback control started after 40 min at 10 °C in experiment 1 and after 10–15 h at the same temperature in experiment 2. Figure S8 shows the results for experiment 1: the temperature did not converge to 10 °C while keeping the counts at the setpoint of 1000 total counts/measurement The amplitude of the cycles kept decreasing with time while the lowest temperature for each cycle increased, as shown in Figure S8a. The solute concentration inferred from the Raman signal is shown in Figure S8b. For this experiment the temperature could not converge to its final setpoint and cycles became smaller and smaller while keeping a constant higher temperature of around 40 °C. After two days from the beginning of the experiment, the temperature was forced to decrease to 10 °C generating secondary nucleation, as shown by the increase in the number of counts/meas. of Figure S8a and the high amount of fines at the end of the experiment (see Figure S8a and S9). Figure S8c and

S10 show microscopic images of the crystals during cycling that indicates how this strategy promoted de-agglomeration of crystals. However, the DNC strategy could not converge to the final temperature, resulting in poor recovery yield. A possible explanation for this behavior is that temperature cycling started at relatively high supersaturation as it can be noted in Figure S8b. At this high supersaturation the monohydrate crystals were too small in size and still had very high tendency to agglomeration. Such agglomerates would de-aggregate during heating and then aggregate again while cooling with minimal growth, especially at high supersaturations.

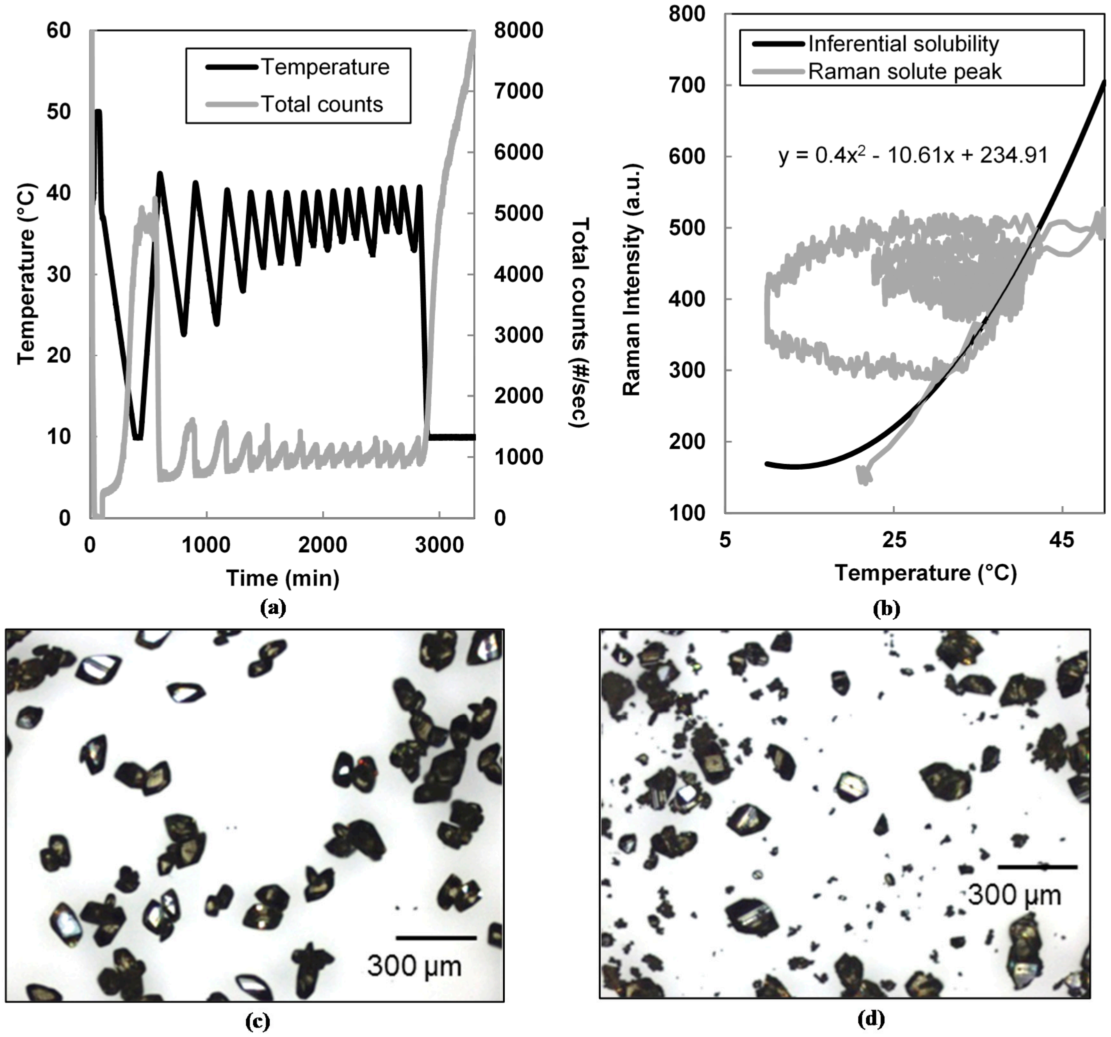

Figure S8. (**a**) Temperature and total counts during the direct nucleation control (DNC) experiment 1. (**b**) Raman intensity of the solute peak as a function of temperature during the experiment. (**c**) Microscopic image of crystals during cycling (2000 min). (**d**) Microscopic image of crystals at the end of the experiment. The interplay between agglomeration/de-agglomeration, secondary nucleation and growth for this specific experiment determined the oscillatory trend shown in Figure S8a. In order to avoid this phenomenon a different approach was used in the DNC experiment 2.

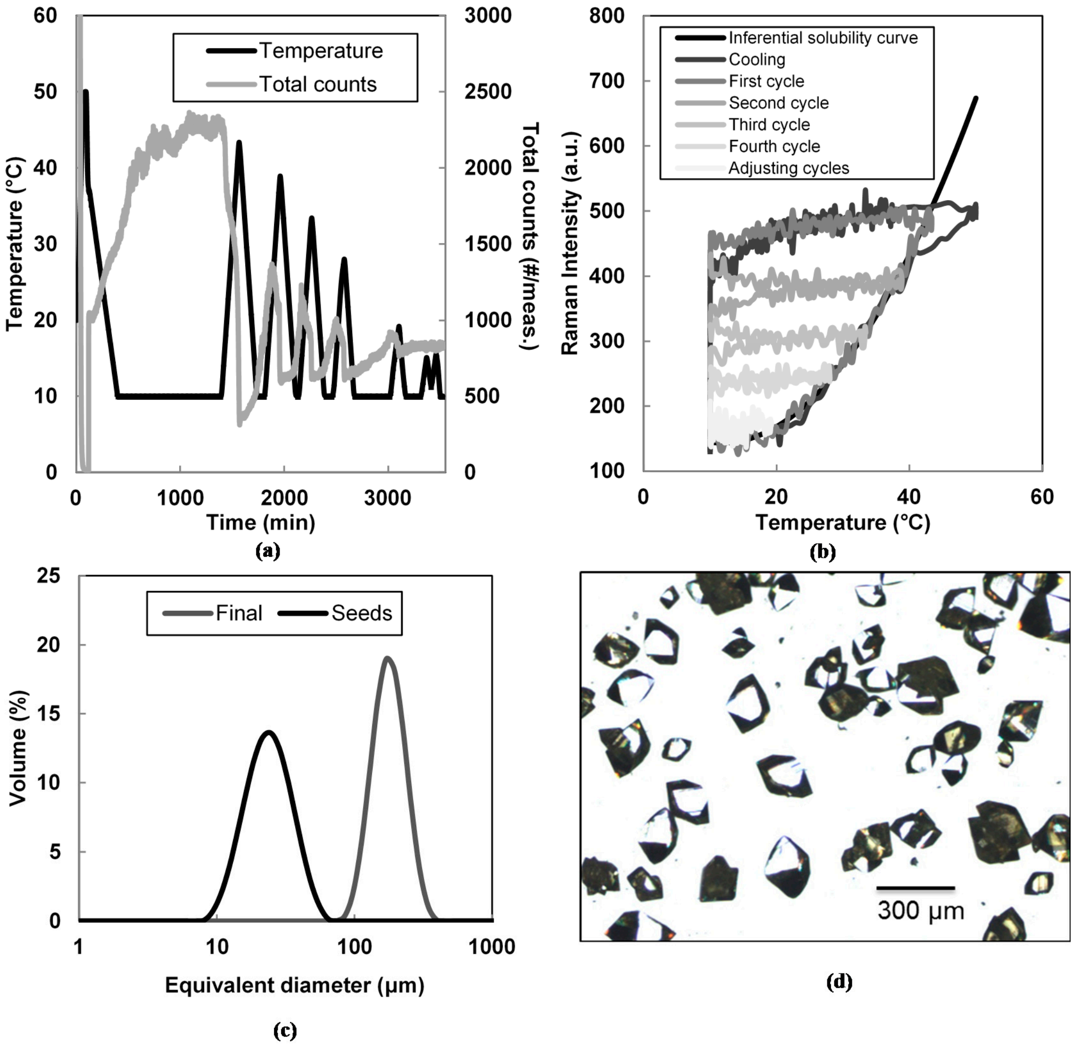

Figure S9. (**a**) Temperature and total counts during the DNC experiment 2 (**b**) Raman intensity of the solute peak as a function of temperature during the experiment (**c**) Initial and final crystal size distribution from Malvern Mastersizer (**d**) Microscopic image of the crystals at the end of the experiment.

In this experiment, after seeding and cooling, the temperature was kept at 10 °C for 10–15 h before starting the DNC. In this way the monohydrate crystals and agglomerates were allowed to grow to a larger size compared to experiment 1 and agglomeration/de-agglomeration during cycling was avoided. As shown in Figure S9b, all the supersaturation was consumed for crystal growth, secondary nucleation and agglomeration after cooling down to 10 °C.

The following four cycles dissolved the finer particles and destroyed agglomerates, the highest temperature reached for each of them decreases over time, while the lowest is always around 10 °C. Differently from DNC experiment 1, the temperature cycles converged to a low temperature while keeping the counts/meas. at the desired setpoint of 850 total counts/measurement.

Five main cycles plus two adjusting ones are generated by the feedback control during the experiment. The final solute concentration corresponds to the equilibrium value at 10 °C and crystals after filtration and drying do no show significant agglomeration (see Figure S9d and S11). Figure S9c and show the CSD evolution during the experiment. Crystals grew from a volume weighted mean diameter of 25 μm to 183 μm (+627%) and the span of the CSD decreased from 1.03 to 0.73 (−29%) as a result of fines removal (see Table S1 for the Mastersizer data). DNC allowed reaching a larger crystal size compared to the seeded temperature cycling experiments while promoting de-agglomeration

and allowing a reasonable yield.

Table S1. Malvern Mastersizer data for the seeds and the crystals at the end of the DNC experiment 2, where d(0.1), d(0.5), and d(0.9) are the particle diameters at 10%, 50% and 90% of the cumulative distribution, Span was calculated as [d(0.9) – d(0.1)] / d(0.5), and D[4, 3] and D[3, 2] are the volume and surface weighted mean diameters, respectively.

Sample	d(0.1) (μm)	d(0.5) (μm)	d(0.9) (μm)	Span (–)	D[4, 3] (μm)	D[3, 2] (μm)
Membrane seeds	14.2	23.5	38.5	1.03	25.2	21.8
Final DNC 2	124	176	252	0.73 (−29%)	183 (+627%)	170 (+678%)

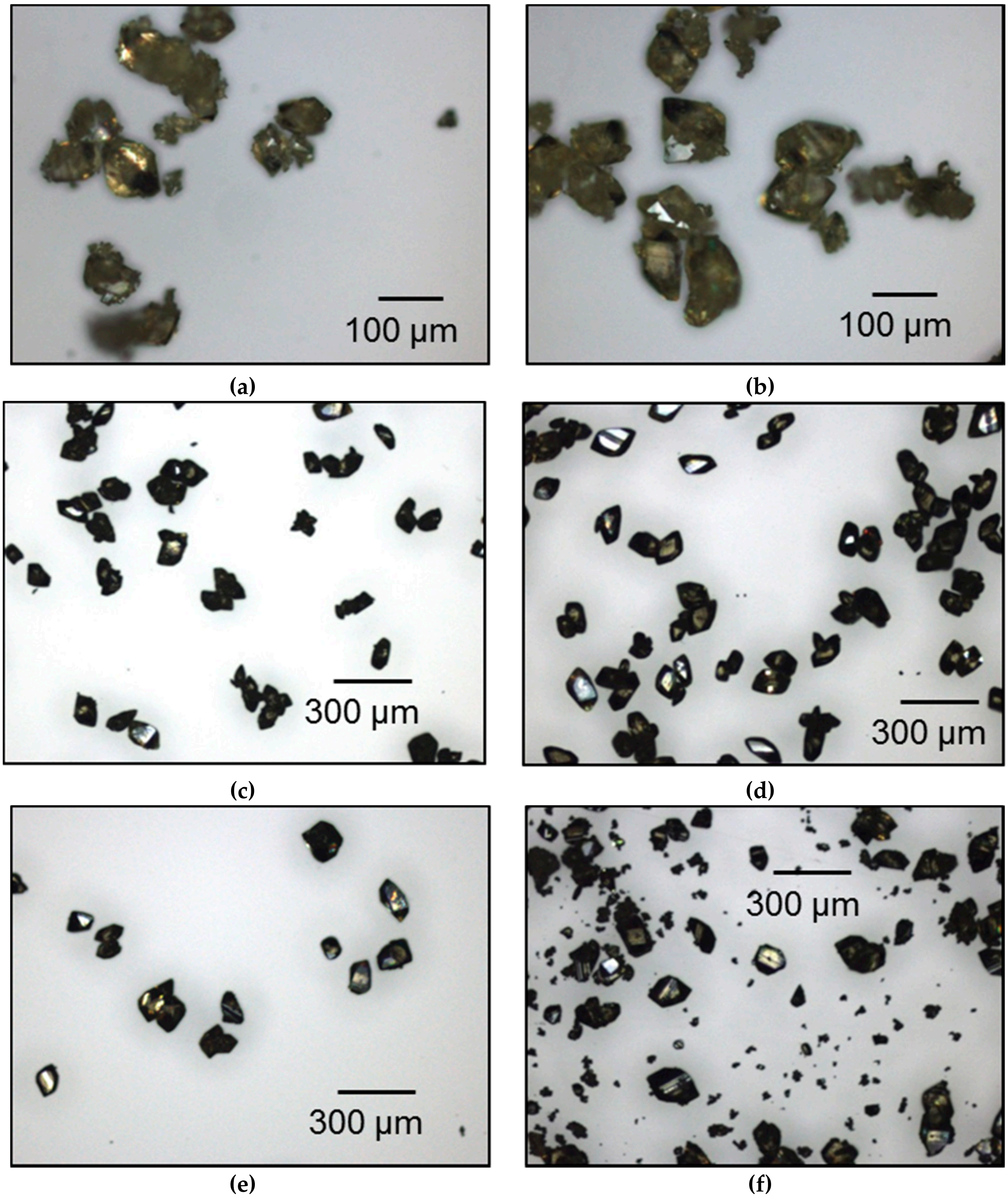

Figure S10. Microscopic images of crystals during the DNC experiment 1 (not converging, setpoint 1000 total counts/measurement) taken at times (**a**) 385 min (**b**) 555 min (**c**) 1375 min (**d**) 2830 min (**e**) 2880 min (**f**) 3300 min.

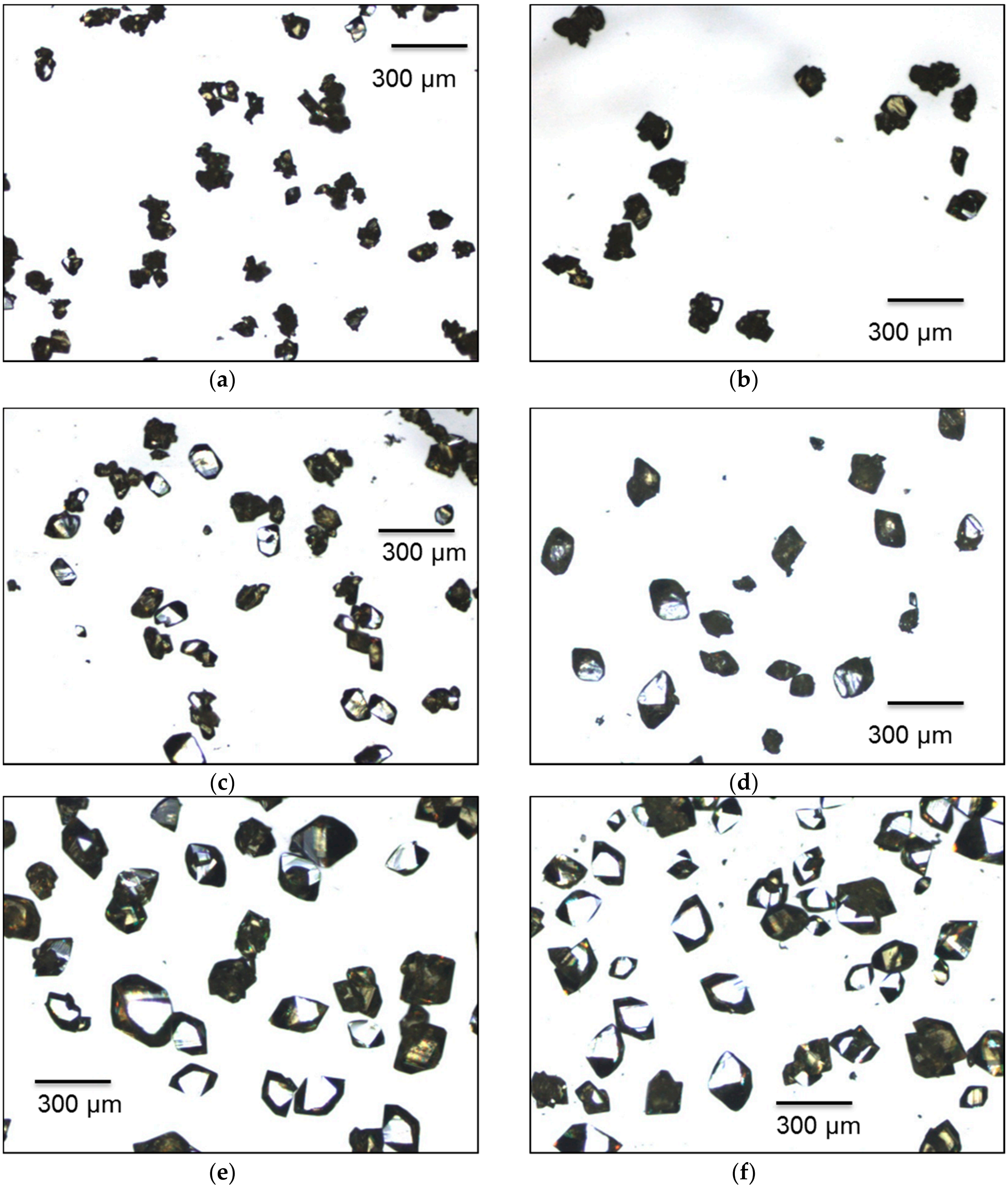

Figure S11. Microscopic images of crystals during the DNC experiment 2 (converging, setpoint 850 total counts/measurement) taken at times (**a**) 435 min (**b**) 1385 min (**c**) 1715 min (**d**) 1965 min (**e**) 2825 min (**f**) 3525 min.
